# Sonoelectrochemical Synthesis of Nanoparticles

**DOI:** 10.3390/molecules14104284

**Published:** 2009-10-23

**Authors:** Veronica Sáez, Timothy J. Mason

**Affiliations:** Sonochemistry Centre, Faculty of Health and Life Sciences, Coventry University, Priory Street CV1 5FB, Coventry, UK

**Keywords:** electrodepostion, nanoparticles, sonoelectrochemistry, sonochemistry

## Abstract

This article reviews the nanomaterials that have been prepared to date by pulsed sonoelectrochemistry. The majority of nanomaterials produced by this method are pure metals such as silver, palladium, platinum, zinc, nickel and gold, but more recently the syntheses have been extended to include the preparation of nanosized metallic alloys and metal oxide semiconductors. A major advantage of this methodology is that the shape and size of the nanoparticles can be adjusted by varying the operating parameters which include ultrasonic power, current density, deposition potential and the ultrasonic vs electrochemical pulse times. Together with these, it is also possible to adjust the pH, temperature and composition of the electrolyte in the sonoelectrochemistry cell.

## 1. Introduction

Nanomaterials have broad applications in a variety of fields because of their unusual and size dependent optical, magnetic, electronic and chemical properties [1,2]. Nanoparticles are characterized by an extremely large surface to volume ratio, and their properties are determined mainly by the behaviour of their surface [3,4]. The applications of nanoparticles are well known in the fields of cosmetics and pharmaceutical products, coatings, electronics, polishing, semiconductors and catalysis, and the design and preparation of novel nanomaterials with tunable physical and chemical properties remains a growing area. There are a range of methods of producing metallic nanosized materials including radiation methods [5], thermal decomposition [6], vapor deposition [7], reduction in microemulsions [8] and chemical reduction methods [9]. However most of these techniques tend to be expensive and time-consuming. An alternative method, simple and cost effective, is the use of sonoelectrochemistry [10].

The application of ultrasound irradiation to electrochemistry processes date back to the 1930s [11]. However, in the last decade the expansion of the sonoelectrochemistry has become increasingly important [12]. The variety of induced effects on electrochemistry processes by ultrasound waves can be attributed to the generation, growth and collapse of microbubbles in the electrolyte [13]. If the cavitation takes place close to the surface of the electrode, a jet of liquid penetrate inside the bubble perpendicular to the electrode surface leading to the formation of high velocity microjet of liquid toward the surface [14]. When the ultrasound intensity is higher than the threshold intensity, the collapse of the bubbles is also associated with shock waves [15] and microstreaming [16]. All these phenomena lead to the decreasing of the diffusion layer thickness [17,18] and can improve the overall mass transport, increasing reaction rates, indeed of cleaning and degassing of the electrode surface [19,20]. Chemical effects [21,22] associated with the generation of radical from the sonolysis of the solvent also were observed. Recently there is a growing interest of the application of the sonoelectrochemistry in environmental remediation [23,24] and in the preparation of nanopowders [25].

A number of different arrangements of equipment have been used for the introduction of the ultrasound irradiation into the electrochemical systems. The first and simplest setup used was the immersion of a conventional electrochemistry cell into a fixed position in the ultrasound bath [13]. Some studies were carried out using this configuration [26] but the power transmitted inside the electrochemistry cell is low and the results depend strongly on its positioning because the distribution of the ultrasound field is not homogeneous [27]. Another arrangement is the introduction of an ultrasonic horn system (often referred to as an ultrasonic probe) directly into an electrochemistry cell. This allows the ultrasonic waves to be directed onto the electrode surface and provides much more efficient power control. Various types of sonoelectrochemistry cells using ultrasound probes have been reported. In the most used configuration the electrodes and the ultrasound horn are immersed in the solution with the ultrasound horn emitter is placed face to face at a known distance from the electrode surface [13]. Another arrangement is to convert the ultrasound horn itself into the working electrode [28]. Such an electrode is referred to as sonotrode [13] or sonoelectrode [28]. This new type of sonoreactor was first introduced by Reisse *et al.* [28] to study the electrodeposition of copper and the electroreduction of benzaldehydes [29,30] and benzoquinone [30] have also been reported using this device. Subsequently the sonotrode system involving consecutive pulses of electrolysis (for deposition) and ultrasound (to release the deposit) has been used in the preparation of nanopowders.

This review is focused on the preparation of nanomaterials using the pulsed sonoelectrochemistry method. The first part summarizes the basic principle of this methodology and the different materials including metals, alloys, semiconductors and conductive polymers that have been prepared to date while the second part examines the effect of the different variables on the process.

## 2. Experimental Devices and Methodology

**Figure 1:**
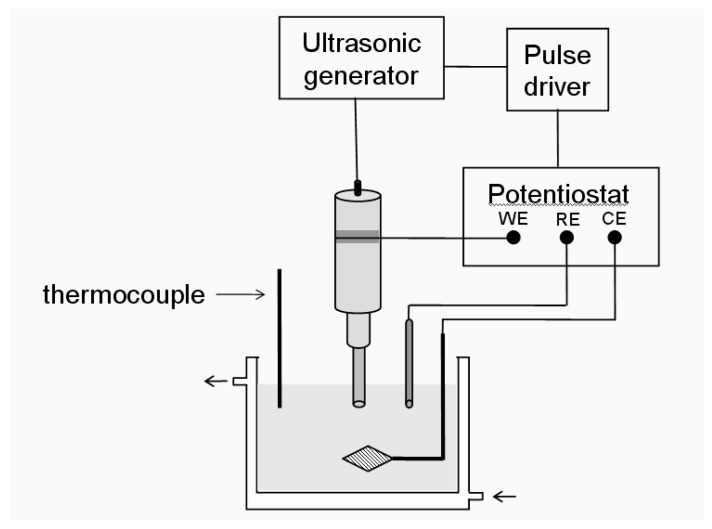
Sonoelectrochemistry set-up used in the production of nanopowders (WE working, RE reference and CE auxiliary electrode).

Reisse *et al.* have described a device for the production of metal powders using pulsed sonoelectrochemical reduction [28,31]. Figure 1 shows the experimental set-up used. In these experiments a titanium probe (20 kHz) acts both as a cathode and an ultrasound emitter. The electroactive part of the sonoelectrode is the planar circular surface at the bottom of the horn and the immersed cylindrical part into the electrolyte is covered by an isolating plastic jacket. The ultrasound probe is connected to a generator and a potentiostat using a pulse driver.

The first system used the simplest configuration of a two-electrode cell because the process is carried out under galvanostatic conditions. The drawback of this configuration is the presence of undesirable secondary reactions under galvanostatic control and to overcome this, an adaptation was made. The replacement of a two-electrode configuration (cathode and anode) by a three-electrode configuration (working, reference and auxiliary electrodes) [32] in the sonoelectrochemistry system was carried out with the aim of applying a controlled potential to the sonoelectrode to get a better control of the process. In the majority of cases the processes have been carried out under galvanostatic conditions because using this configuration is simpler and it could be used for the large scale production of nanoparticles.

It is worth mentioning that before any sonoelectrochemistry experiment the ultrasound power delivered inside the cell should be measured using calorimetric methods [33]. The fundamental basis of the pulsed sonoelectrochemical technique for the production of nanopowders is massive nucleation [34]. At the cathode, a pulse of current (or potential) reduces a number of cations, depositing a high density of metal nuclei on the sonoelectrode surface, and the titanium horn works only as an electrode during this time (T_ON_). This short electrochemical pulse is immediately followed by a short pulse of high intensity ultrasound (T_US_) that removes the metal particles from the cathode surface and replenishes the double layer with metal cations by stirring the solution. Sometimes, a rest time (T_OFF_), without current or ultrasonic vibrations, follows the two previous pulses and it is useful to restore the initial conditions close to the sonoelectrode surface.

**Figure 2:**
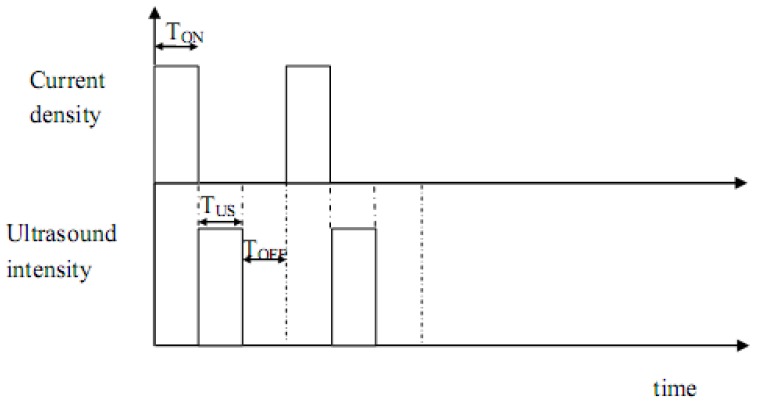
Distribution of the ultrasound and current pulses with the time (T_ON_ current pulse time, T_US_ ultrasound pulse time and T_OFF_ rest time).

Figure 2 shows the distribution of the pulses with the time. Electrochemical and ultrasound pulses typically ranges between 100 and 500 ms and the rest time lasts no more than 1 s.

In most commercial systems the ultrasound horns are made from titanium alloy (Ti:Al:V 90:6:4). In air, titanium forms a surface oxide layer, consisting of a mixture of TiO_2_, Ti_2_O_3_ and adsorbed oxygen. Under an oxidation process a passivated film can grow on the sonoelectrode surface and this acts as an insulator [13,35]. This limitation restricts the use of the sonoelectrode to reduction process. Before each experiment, the titanium sonoelectrode should be polished [35] as a normal electrode used in electrodeposition process, to remove any contamination on the surface that could affect to the process of nucleation.

This new electrochemical method has since been employed to produce numerous pure metals [32,36,37] or alloys nanopowders [38,39] and semiconductor nanoparticles [40]. More recently, conductive polymer nanoparticles [41] have also been synthesized by pulsed sonoelectrochemistry. The metal powders obtained are in a finely divided state with high surface areas, an average particle size of 100 nm and high chemical purity [42]. Table 1 summarizes the experimental conditions used in the synthesis of different materials by pulsed sonoelectrochemistry.

## 3. The Preparation of Nanoparticles by Pulsed Sonoelectrochemistry

### 3.1. Metallic Nanopowders

Copper electrodeposition is a well-known industrial process where all electrodeposition parameters and electrolytes are well established [43], thus copper was one of the first metals to be synthesized using pulsed sonoelectrochemical methods. The synthesis of a range of metallic copper nanostructures by pulsed sonoelectrochemistry has been reported [28,31,44,45]. Haas *et al*. [44] synthesized copper nanoparticles from an aqueous acidic solution of CuSO_4_ using polyvinylpyrrolidone (PVP) as a stabilizer. By applying a range of current density between 55 and 100 mA cm^-2^ monodispersed spherical copper nanoparticles with a diameter range of 25-60 nm were observed. A reaction mechanism between copper ions and PVP was proposed. The first step was the formation of a coordinative bonding between PVP and copper ions, forming a Cu^2+^-PVP complex. The formed complex was present in the solution and when the current pulse was applied, the Cu^2+^ was reduced to Cu^0^ on the polymer preventing the agglomeration of the metallic nanoparticles. IR studies showed that the PVP is coordinated with Cu through C-N and C=O bonds. When PVA was used as a stabilizing agent [45] copper with dendritic morphologies was obtained.

Zin *et al.* [10] reported the production of platinum nanoparticles from aqueous chloroplatinic solutions under galvanostatic conditions. The platinum nanoparticles produced were spherical with an average size ranging from 10 to 20 nm. These aggregated into secondary structures with a mean size ranging between 100 and 200 nm. Tridimensional dendritic Pt nanostructures [37] were synthesized when PVP was used as stabilizer.

Stable suspensions of gold nanoparticles in water have been reported by Aqil *et al.* [32] using pulsed sonoelectrochemistry. Some polymers were added to the electrolyte to avoid the nanoparticle aggregation that is frequently observed in such sonoelectrochemistry techniques [31]. The gold electrodeposition was carried out applying a potential in the range of −850 to −1,300 mV vs NHE and in the presence of α-methoxy-ω-hydroxyl polyethylene (MPEO) the nanoparticles aggregated and settled down in the electrochemical cell in a similar way to that observed without stabilizer present. However using a MPEO/PVP polymer mixture, a stable violet suspension was obtained at the end of the process without sedimentation. This sample showed a narrow size distribution centered on 12 nm, together with a few larger particles (30 nm). In the presence of polyethylene oxide (PEO) disulfide polymer a very stable suspension of gold nanoparticles with a mean diameter of 35 nm was obtained. Table 2 summarizes the different strategies used to prepare stable suspensions of gold nanoparticles and the average size obtained.

Silver nanoparticles have also been synthesized using different electrolytes and stabilizers by the pulsed sonoelectrochemistry method [46,47,48,49]. Shaped silver nanoparticles including spheres, rods and dendrites were prepared from an aqueous solution of AgNO_3_ in the presence of nitriloacetate (NTA) [46]. It was found that the electrolyte composition come along reaction time can greatly affect the shape and growth of the nanoparticles. Without NTA, shaped Ag nanoparticles were not formed. If the concentration of NTA is very low (less than 0.1 g/L) only randomly shaped aggregates were obtained but increasing the concentration of NTA up to 1 g/L the formation of the shaped particles was favored. Using this stabilizer concentration and varying the concentration of silver ions in the electrolyte, silver nanoparticles were formed with different shapes. With 0.6 g/L AgNO_3_ the nanoparticles were spherical and well dispersed, with a size of 20 nm with a reaction time below 5 min. Nanorod structures with a width of 10-20 nm in diameter were obtained with 2 g/L AgNO_3_ after 12 minutes of reaction and when the concentration of AgNO_3_ increases to 20 g/L, highly ordered dendritic nanostructured were observed for a 25 min reaction. The authors suggested that it may be that the excess of silver ions in the solution that favors the aggregation and growth into the dendritic structure of Ag clusters. In subsequent work Socol *et al.* [47] used the same system, silver + NTA and proposed a model based on a suspensive electrode formation to explain the growth of dendritic structures. The model involves expulsion of the particles from the electrode surface as a result of the ultrasonic pulse into the solution where they can remain suspended. These suspended nanoparticles are then moved about in the solution through the influence of the ultrasonic wave and as a result could hit the electrode and thus acquire a charge. These charged particles travelling into the bulk solution could reach the anode and initiate electrodeposition onto themselves and grow in size into a dendritic structure.

Jiang *et al*. [49] reported the synthesis of silver nanoparticles with a face-centered cubic structure in a saturated solution of silver citrate in the presence of PVP. Under the experimental conditions used the Ag nanoparticles were prepared as spherical and monodisperse with an average size of 20-25 nm. In contrast, amorphous silver nanoparticles [48] of 20 nm size were prepared from an aqueous solution of AgBr in the presence of gelatin.

Lei *et al.* [50] reported the synthesis of spherical nanoparticles of tungsten by pulsed sonoelectrochemistry. The average diameter of these nanoparticles was 30 nm and some aggregated particles were observed. Qui *et al.* [36] synthesized highly dispersed palladium nanoparticles by pulsed sonoelectrochemistry methods with different sizes and shapes using a solution of PdCl_2_ in the presence of cetyltrimethylammonium bromide (CTAB). The nanoparticles were mostly spherical with an average size of 4–5 nm and for longer reaction time than 2.5 h, dendritic structured Pd was detected. The dendritic palladium was made up of numerous spherical Pd nanoparticles with a diameter of approximately 10 nm. Zinc [31,42,51], nickel [42,51] and cobalt [42] nanoparticles have also been successfully synthesized by pulsed sonoelectrochemical methods.

Nanoparticles of very reactive metals with a high negative reduction potential, e.g. magnesium and aluminum can also be synthesized using pulsed sonoelectrochemistry. The synthesis of Mg nanoparticles [52] was carried out using two different electrolyte solutions based on Grignard reagents (EtMgCl and BuMgCl) in ethers. The ethers used were tetrahydrofuran (THF) and dibutyldiglyme (DBDG) and AlCl_3_ was added to increase the conductivity of the electrolyte. Four nm sized metallic magnesium particles were obtained for both electrolytes. The product efficiencies in THF and in DBDG were different at 41% and 33%, respectively, and this was ascribed to the difference in solution viscosity. MgO nanoparticles could also be found in the product due to the ease of oxidation of the metal.

In the case of aluminum nanoparticles [53] a solution of LiAlH_4_ and AlCl_3_ in THF was used. TEM analysis showed the formation of aggregated particles in the range 10–20 nm and Energy-dispersive X-ray (EDX) analysis confirmed that the surface of the material is mainly composed of aluminum but there were also small peaks observed for oxygen which showed that the surface of the aluminum was partially oxidized. This was a similar result to that obtained for the magnesium nanoparticles. The current pulse used in the synthesis of aluminium and magnesium nanoparticles was 600 s which is much longer than those commonly used in the other nanometal syntheses.

### 3.2. Alloy Nanopowders

The pulsed sonoelectrochemistry technique has been applied to the synthesis of binary and ternary alloyed nanopowders containing iron, cobalt and nickel [25,38,39]. All these alloys were synthesized using an electrolyte bases on Aotani’s formulation. Binary and ternary alloys were deposited galvanostatically at 8,000 A m^-2^ and particles with a mean diameter of 100 nm were produced [25]. In a recent report a series of iron-cobalt alloy nanopowders were prepared by pulsed sonoelectrochemistry under different potentiostatic conditions [38]. Under these experimental conditions the composition of the alloys is essentially independent of the deposition potential and is only determined by the composition of the electrolytic bath. The smallest particles detected had a mean diameter of 7 nm and were strongly aggregated in three-dimensional clusters.

Dabala *et al*. [39] calculated the cathode efficiency for production of Co_65_Fe_35_ nanoparticles as the ratio of the produced mass of powders to the Faradaic yield. For applied currents lower than -20 mA the efficiency was high and remained over 50% but as the applied current increased 50-fold, the cathode efficiency decreased by 10-fold.

### 3.3. Semiconductor Nanopowders

Semiconductor materials are the foundation of modern electronics and are finding applications in photochemistry, dye-sensitized solar cells, and in the photocatalytic treatment of chemical waste [54,55].

Cu_2_O nanopowders have been prepared in potentiostatic mode [56]. The work was based on a previous voltametric study which showed that at an applied potential ranged between −0.65 and −1 V/SSE it was possible to avoid the formation of a mixture of Cu_2_O and Cu. The powders generated were analyzed by XRD and only Cu_2_O peaks were detected indicating that neither metallic copper nor CuO were formed at these potentials. TEM micrographs showed numerous agglomerates of nanoparticles of a variety sizes but isolated particles were also found with diameter ranges between 7 and 20 nm.

Shen *et al*. have reported the synthesis of CdSe with a tubular structure [57]. This synthesis was carried out by applying a constant current density in the range of 60-80 mA cm^-2^, but in this case under continuous sonication. The CdSe nanotubes had an outer diameter of 80 nm and a wall thickness of 10 nm and were obtained through a roll-up mechanism. In the first stage 2D nanosheets were formed on the electrode and these were then dislodged from the sonotrode surface. Due to the high surface energy of the edges of the nanosheets, these flexible and unstable nanosheets are thought to roll-up and form tubular nanostructures during the cavitation process.

PbTe nanorods [40] were synthesized by pulsed sonoelectrochemistry methods using NTA as stabilizer. PbTe formation predominated when the Pb^2+^/NTA ratio was high, even at a very low concentration of Te^2-^ ions. However, for low Pb^2+^/NTA ratio, the thermodynamic solubility constant limited the precipitation of PbTe even at relatively high concentrations of Te^2-^ ions. With very low concentrations of Pb^2+^ (2mM) only spherical particles were observed and the increase in the concentration of Pb^2+^ ions caused the spheres to grow and rod-shaped morphologies to be formed. PbTe nanorods showed highly uniform nanorod morphology with an average diameter of 7 nm.

Other semiconductor materials as PbSe [58], Bi_2_Se_3_ [59], and MoS_2_ [60] have been successfully synthesized with different morphologies by using the pulsed method in aqueous solution at room temperature.

### 3.4. Conducting Polymer Nanoparticles

Polyaniline (PANI) and other conducting polymers such as polythiophene, polypyrrole, and poly(methylaniline) have great potential in numerous technological applications [61]. These materials can exist as bulk films or as dispersions but a common problem with the latter is particle aggregation which limits the range of applications. Conducting-polymer synthesis using pulsed sonoelectro-chemistry has been reported [41,62] despite the fact that oxidation of the horn surface can generate an insulating layer when the sonoelectrode is used in oxidation process [13,35]. Ganesan *et al*. [62] reported the synthesis of PANI nanomaterial by oxidative polymerization using the sonoelectrode as anode. In this synthesis a constant potential pulse of +1V vs Ag/AgCl/3M NaCl was applied to the sonoelectrode in an aqueous solution of aniline in HCl. After 2 h, the formation of PANI nanostructures was confirmed by UV-Vis and TEM micrographs showed particles with a diameter of 2-4 µm made up of very small nanoparticles of average size 20-40 nm. 

Mahito *et al.* [41] reported the synthesis of nano poly(methylaniline) by the use of a pulsed sonoelectrochemical method, but in this case the anode was a platinum electrode placed face to face with the ultrasound emitting surface of a horn. This arrangement overcame the drawback related to the use of an ultrasound horn in oxidation process. Poly(methylaniline) synthesis was achieved at a constant potential pulse of 0.75 V vs. SCE to the platinum electrode in an aqueous solution of methylaniline in HClO_4_. PNMA microspheres were obtained and their size distribution depended on the electric pulse width. Thus for a pulse width of 40 s the average size was 1.4 µm, whereas for 90 s the average size was 2.4 µm.

## 4. Parameters that Control the Formation of Nanoparticles

There are many experimental variables involved in the formation of nanoparticles via the sonoelectrochemical route in terms of particle size and process efficiency. Some of these are discussed below together with possible explanations:

### 4.1. Bath Temperature

Crystal growth is slower at low temperatures, resulting in smaller crystal size at these temperatures [63]. On the other hand at higher temperatures the quantity of powders obtained is very low. The latter can be ascribed to the increased rate of re-dissolution of the nanoparticles with increase in the temperature. In general therefore to obtain higher efficiencies and small particle size it is necessary to maintain low temperatures in the system.

### 4.2. Current Density

Current density can affect crystal size in at least two opposing directions [58]. At lower currents a smaller size would be expected based on the smaller amount of material deposited however lower currents allow more time for atomic diffusion processes to occur and this can lead to larger crystal size.

**Figure 3:**
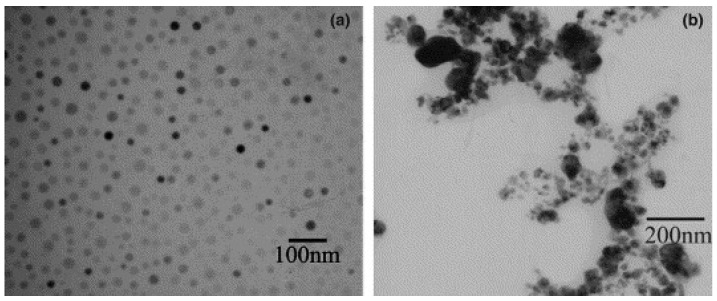
Silver nanoparticles prepared under different current density: (a) 70 and (b) 140 mA cm^-2^. Reprinted from Ref. [49] with permission from Elsevier.

In the synthesis of CdSe [64] the current density was found to be an important factor in that a lower current density resulted in larger crystal size, 10 nm at 100 mA cm^-2^ compared with 5 nm at 250 mA cm^-2^. In contrast to this result the synthesis of copper nanoparticles [44] showed a particle size variation from 29 to 10 nm when the applied current density increased from 55 to 100 mAcm^-2^. With the exception of a few reports [49,64] (Figure 3) where an increase of the current caused a larger crystal size or even aggregated particles most authors concluded that raising the current density resulted in smaller nuclei and faster nucleation [36,37,44].

The current density has also been found to affect the yield of the process since at high current density secondary reactions can take place decreasing the yield of the nanoparticles. One such undesirable secondary reaction is the reduction of water and is the main cause of low cathode efficiencies. Thus in the production of Co_65_Fe_35_ alloy nanoparticles [39] cathode efficiency remained over 50% for applied currents lower than -20 mA but as the applied current increased by 50-fold, the cathode efficiency decreased 10-fold, due to the reduction of water as evidenced by hydrogen evolution in the sonoelectrode.

### 4.3. Current Pulse Time (*T_ON_*)

The process requires that the deposit should be removed during the period of each sonic pulse and therefore only new nuclei should be formed during T_ON_. As the length of T_ON_ increases so the formation of a stable metallic nucleus on the sonoelectrode and the growth of the formed nuclei will take place. Therefore, in principle, to obtain a smaller crystal size a short *T_ON_* is required. It has been observed experimentally that shorter current pulses increased the process efficiency [39]. However in the synthesis of Pt nanoparticles [10] T_ON_ was varied between 200-500 ms without any significant effect on either the efficiency of the process or the nanoparticles size. Also in Cu_2_O nanoparticle synthesis the particle size did not vary significantly when T_ON_ ranged between 100 and 300 ms [56].

### 4.4. Ultrasound Intensity

The intensity of the ultrasonic pulse should be high enough to remove the whole deposit from the sonoelectrode surface and not to leave any residue on the surface of the working electrode preventing by this way the growth of the metal particles during the next current pulse [31]. Above this value of intensity where the deposit is removed, further increase in intensity is not expected to affect crystal growth significantly [34] and the extra energy will be wasted. However it should be noted that the ultrasound pulses can affect the morphology and size of the freshly formed nanopowders in the solution. Suslick [65,66] has shown that for ultrafine nickel powders ultrasonic irradiation causes rapid particle movement in the bulk solution resulting in high velocity collisions resulting in the formation of agglomerates.

### 4.5. Ultrasound Pulse Time (T_US_)

As stated above the ultrasonic pulse time (*T_US_*) must be sufficient to remove all metal crystallites that are deposited on the sonotrode during electrodeposition. In Pt nanoparticle synthesis *T_US_* was varied between 300 and 500 ms [10] and although the efficiency decreased at higher *T_US_* the particle size remained constant. Also Cu_2_O nanoparticle size did not vary significantly with *T_US_* between 100 and 400 ms [56]. This lack of dependence of diameter with *T_US_* could be explained by the fact that there is a sufficient effective ultrasonic pulse time so that all of the particles are expelled away from sonoelectrode surface well before the end of the pulse time. It is interesting to note that sonoelectrodeposition under continuous ultrasound irradiation leads to the formation of amorphous material [34].

### 4.6. Stabilizer

**Figure 4:**
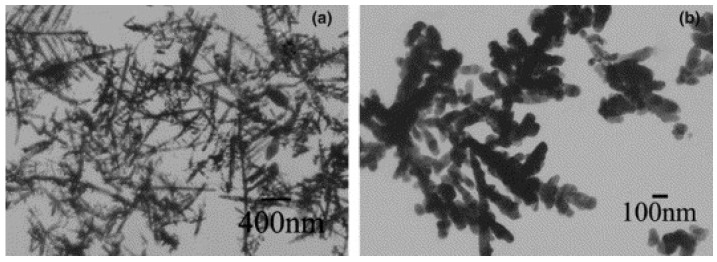
Silver nanoparticles prepared (a) without PVP and (b) 0.1 g/l PVP. Reprinted from ref [49] with permission from Elsevier.

In pulsed sonoelectrochemistry the nanoparticles initially formed have pure uncontaminated surfaces. Such powders are so fine that they can agglomerate easily e.g. by impact with the wall of electrochemical cell. The acoustic streaming associated to the propagation of ultrasound wave in the solid suspension is also sufficient to induce the agglomeration process [31]. Several authors have used polymer stabilizers to prevent the agglomeration [32] and to control the shape of the nanoparticles [46,49] (Figure 4).

## 5. Conclusions

Pulsed sonoelectrochemistry techniques, which use 20 kHz ultrasound horn both as working electrode and ultrasound emitter, have been used to prepare nanopowders. The majority of nanomaterials produced by this method are pure metals. More recently the syntheses have been extended to include the preparation of nanosized metallic alloys, metal oxide semiconductors and conductive polymers. Factors which affect the process yield and particle size are the ultrasound pulse time and the current density. In general, decreasing temperature, shorter pulse duration, high current density and high ultrasound intensity will lead to a reduction in crystal size. These parameters need to be optimized in order to maximize the nanoparticles production yield and to obtain the lowest size of the products depending of the applications.

Nanomaterials that have been prepared to date by pulsed sonoelectrochemistry have been reviewed in this article. Nanoparticles aggregated were formed but the shape and size of the nanoparticles can be adjusted by controlling the various electrodeposition and ultrasound parameters and using the suitable stabilizer.

## Figures and Tables

**Table 1:** Experimental conditions for the pulsed sonoelectrochemistry synthesis for different nanomaterials using a 20kHz titanium horn as working electrode.

Species	Solution	I_US_/W cm^-2^	T_US_/ms	Electrochemistry conditions	T_ON_/ms	T_OFF_/ms	Experiment duration	Size	Ref.
Cu	0.16 mol/L CuSO_4_ 5H_2_O, 1.84 mol/L H_2_SO_4_, pH 0.5	62	100-600	440-480 mA cm^-2^	250-900	150-300	30 min	With PVP 29-34 nm Without PVP 200 nm aggregates	[44]
Pt	0.1M K_2_PtCl_4_, 0.5M NaCl pH 1	62	300-500	50 mA cm^-2^	200-500	-----	1h	10-20 nm (some aggregated 100 and 200 nm)	[10]
Au	2.8 10^-4^M HAuCl_4_·nH_2_O 1g/L MPEO pH 1	Not indicated	100	-850 to -1300 mV/ NHE	10-50	100-200	5h	5 -35 nm	[32]
Mg	Grignard reagents (EtMgCl and BuMgCl), AlCl_3_ in THF and DBDG	62	300	5 mA cm^-2^	6 10^5^	600	NI	4.5±0.5 nm	[52]
CdSe	CdCl_2_ 2.5H_2_O, NTA, Na_2_SeO_3_ with PVP	NI	Cont.	60-80 mA cm^-2^	Cont.	----	2h	80 nm diameter nanotubes	[57]
Co_65_Fe_35_	Sulphate bath based on Aotani’s formulation pH 3	62	300-500	8-380 mA cm^-2^	300-500	Not used	90 min	3-D structures 300 nm	[39]
PANI	0.5M aniline, 0.5M HCl	62	NI	+1V/ Ag/AgCl (3M)	8 10^6^	800	2h	20-40 nm	[62]
Cu_2_O	0.45 mol L^-1^ CuSO_4_ 5H_2_O + 3.25 mol L^-1^ lactic acid pH 9.1	110	100-400	-0.65, -1.2V/ SSE	100-300	200-400	NI	8 nm	[56]

Abbreviations: NI = Not indicated; Cont. = Continuous.

**Table 2:** Summary of experimental conditions in gold nanoparticles synthesis. Adapted from Ref. [32].

Polymer	E/mV vs ENH	T_ON_/ ms	Size/ nm
MPEO	−850	50	Sediment
MPEO/PVP	−850	50	12
PEO disulfide	−1,300	20	35
